# Evaluating and Improving Light Absorption Retrievals of Black Carbon Using In Situ Polar Nephelometry

**DOI:** 10.1021/acs.est.5c05919

**Published:** 2025-09-25

**Authors:** Qizhi Xu, Barbara Bertozzi, Robin Lewis Modini, Benjamin Tobias Brem, Thomas Müller, Baseerat Romshoo, Claudia Mohr, Martin Gysel-Beer

**Affiliations:** † 124377 PSI Center for Energy and Environmental Sciences, 5232 Villigen PSI, Switzerland; ‡ 28397 Leibniz Institute for Tropospheric Research, 04318 Leipzig, Germany; § Department of Environmental Systems Science, ETH Zürich, 8006 Zürich, Switzerland

**Keywords:** light scattering, polarimetry, aerosol property retrieval, black carbon aggregate, Multi-Sphere T-Matrix, light absorption, phase function

## Abstract

Black carbon (BC) is among the major contributors to global warming, yet significant uncertainties exist in remote sensing retrievals of BC light absorption. A key issue is the mismatch between the simplified spherical morphology assumption commonly used in these retrievals and the actual fractal-like morphology of BC particles. In situ polar nephelometry provides a unique opportunity to improve these retrieval algorithms. Laboratory-based polarimetric measurements allow for a comparison of retrieved and directly measured properties using independent instrumentation. In our experiments, bare BC aggregates were generated, and phase functions were measured using our newly developed polar nephelometer uNeph. Standard retrievals based on Lorenz-Mie theory poorly reproduced the phase function and polarized phase function of BC, leading to significant bias in retrieved properties beyond the uncertainty of independent measurements. Contrary to previous studies, we demonstrate a good closure between measured and simulated phase functions when using the Multi-Sphere T-Matrix (MSTM) method for BC aggregates in the accumulation size range. BC properties, particularly absorption coefficient and volume concentration, were accurately and precisely retrieved by accounting for the fractal-like morphology. Only two additional parameters were used in MSTM retrieval. This suggests that considering aggregates in remote sensing retrievals under real atmospheric conditions could be feasible.

## Graphical abstract

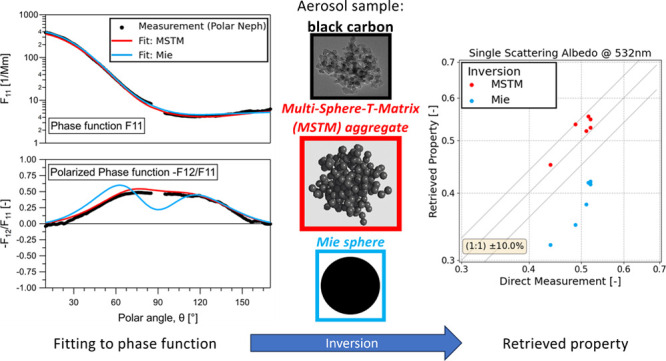

## 1 Introduction

Black carbon (BC) is a side product of incomplete combustion. Here we use the simplified term BC for referring to soot BC, as defined in Corbin et al. (2019), that consists primarily of graphitic sp2-bonded carbon with soot BC particles being aggregates of primary spherules.^1^ BC is the primary contributor to light absorption by aerosols in the visible spectrum,^2^ and it is associated with adverse health effects.^3^ Current global annual BC emissions are estimated at 8 Tg, with approximately 20% from biofuel, 40% from fossil fuel, and 40% from open biomass burning.^4^ In the atmosphere, BC exerts a significant positive climate forcing.^5^ However, the extent of its contribution to warming on global and regional scales remains uncertain.^6–9^ IPCC 2021 ^10^ estimates a range from +0.1 to +0.8 W/m^2^.

Assessing radiative impacts of atmospheric aerosol requires knowledge of columnar extinction of solar radiation (aerosol optical depth, AOD), relative contributions of scattering and absorption to extinction (commonly expressed as single scattering albedo, SSA), in addition to the upscatter fraction, and vertical profile. AOD is directly accessible to ground-based remote-sensing techniques and can be measured with high confidence.^11,12^ By contrast, light absorption and hence SSA are only indirectly accessible through sky radiance measurements combined with polarimetric retrievals.^13^ At present, the aerosol absorption optical depth (AAOD) retrieved from worldwide sun-sky measurements at Aerosol Robotic Network (AERONET) stations is the main product used to evaluate and constrain climate models.^14–16^ Satellite borne multiangle imaging, ideally polarization resolved, serves to constrain aerosol absorption and SSA on a global scale.^17–19^ However, simulated aerosol absorption from most climate models is notably underestimated compared to the values retrieved from remote sensing measurements such as AERONET,^20^ highlighting the need for accurate aerosol remote sensing retrieval products.^11^

Unlike light scattering dominated by bulk aerosol, light absorption in the absence of dust is dominated by BC, for which commonly used Mie-based aerosol models are a poor approximation in terms of mixing state and shape.^21–23^ Combustion-generated BC-containing particles may exhibit a fractal-like geometry resulting from the coalescence and subsequent aggregation of small, nearly spherical, primary particles^24^ within the flame. Understanding the light-scattering behavior of BC aggregates provides the scientific basis for interpreting remote sensing observations of BC.^25^

In situ measurements, known for their high accuracy and comprehensiveness, often serve as benchmarks for validating remote sensing observations and model simulations.^26^ Polar nephelometers have been used for a long time to study the optical properties of aerosol particles.^27–30^ Dolgos et al. (2014) designed and built a laser imaging polar nephelometer called PI-Neph, which can provide intensity and degree of linear polarization of scattered light over a wide range of scattering angles.^31^ Polarimetric data provided by these instruments make it possible to retrieve aerosol properties using inversion schemes similar to those used for remote sensing data retrieval.^32–38^ Schuster^39^ proposed the Statistical Evaluation of Aerosol Retrieval (STEAR), where a retrieval algorithm was evaluated by mimicking atmospheric extinction and radiance measurements using an in situ polar nephelometer in a laboratory experiment. This method was proven to be more robust for determining retrieval algorithm performance than purely theoretical sensitivity studies,^40^ which rely on simplified aerosol optical schemes to compute the scattered radiation fields.

Bare BC particles have a complex fractal-like morphology,^41,42^ and therefore, their polarized phase function differs from that of spherical particles. This presents a potential opportunity to additionally retrieve information on particle shape and to achieve more accurate light absorption retrieval.^43,44^ Simple optical models can offer only limited accuracy and precision when estimating integrated quantities. Lorenz-Mie describes the scattering of an electromagnetic plane wave by a homogeneous sphere which has shown large discrepancies in the results from ambient measurement.^45^ Rayleigh Debye Gans (RDG) theory has been applied to calculate the optical cross sections of fractal aggregates.^46^ However, it assumes the particle’s refractive index is close to the surrounding medium, which is not true for BC, and it also neglects the internal multiple scattering, potentially underestimating scattering and absorption by 30% to 50%.^47,48^ Recently, several studies have moved to more sophisticated optical simulations considering nonspherical geometry by using, e.g., Multi-Sphere T-Matrix (MSTM) or Discrete Dipole Approximation (DDA) algorithms.^48–50^ However, direct comparison between measured and simulated phase function data of atmospherically relevant BC remains very sparse.^51,52^

When it comes to retrieving aerosol properties, a certain level of simplification in representing the aerosol is required to deal with the limited information content of available polarimetric data (also dependent on measurement uncertainties) and the related issue of overfitting.

In this study, we address the trade-off in complexity between an adequate representation of the aggregate geometry of realistic BC particles and the minimal level of detail required for accurate representation of light scattering phase functions to enable accurate retrieval of the effective aerosol geometric and optical properties from polarimetric data. In our experiments, we used nonabsorbing spherical particles, light-absorbing spherical particles, and fullerene soot as test samples. Spherical particles were first used to assess and validate the measurements and a standard retrieval algorithm based on Mie theory. Fullerene soot served as a surrogate for fractal BC aggregates for evaluating the improved performance of retrieval when using MSTM to consider actual morphology. A laser-imaging-type nephelometer (uNeph) was used for polarimetric measurements.^53^ The retrieved aerosol properties were compared with independent measurements conducted alongside uNeph for validation. The primary objective is to demonstrate the feasibility of retrieving volume concentration, particle size, and single scattering albedo for spherical and fractal-like BC particles with high precision and accuracy.

## 2 Materials and Methods

### 2.1 Experimental Setup

#### 2.1.1 Aerosol Generation

As illustrated in Figure 1, aqueous suspensions of insoluble samples or solutions of soluble samples were nebulized by an atomizer aerosol generator (ATM 220, TOPAS, Germany) and then directed through a silica gel diffusion dryer (this together with the use of dry air for diluting the sample further downstream resulted in a relative humidity of less than 10%). The dried aerosol was then brought to charge equilibrium by using a bipolar charger (^85^Kr). In this study, polystyrene latex spheres (PSL, 3000 Series Nanosphere Size Standards Thermo Scientific; insoluble; density 1050 kg/m^3^), nigrosin (Acid black 2, CAS 800-503-6, Sigma-Aldrich; water-soluble; density 1650 kg/m^3^) and fullerene soot (Alfa Aesar, stock no. 40971, LOT no. W08A03, shared by Joshua Schwarz, NOAA; density 1800 kg/m^3^) were used as test aerosols. PSLs are manufactured to uniform properties, spherical shape, and well-defined size, thus making them ideal calibration standards for various instruments, such as particle size analyzers. They are generally considered non-light-absorbing in the visible spectrum and the complex refractive index (CRI) of PSLs is also well-documented by the manufacturer and elsewhere.^54,55^ Nigrosin forms virtually spherical particles through our aerosol generation process.^56^ It is a strongly light-absorbing black dye, whose CRI has also been extensively studied and found to be strongly wavelength- and likely batch-dependent.^56–61^ Fullerene soot is mainly composed of carbon black and a small fraction of fullerenes (around 5%).^62^ It is strongly light absorbing and exhibits an aggregate morphology, as also shown in the transmission electron microscopy (TEM) images in Figure S1. We utilized fullerene soot as a surrogate for BC in our experiments as it allows for testing simulations against measurements across a wide particle size range, although it is known to have a larger primary particle size and to be more compact than fresh diesel soot.^63^

**Figure 1:**
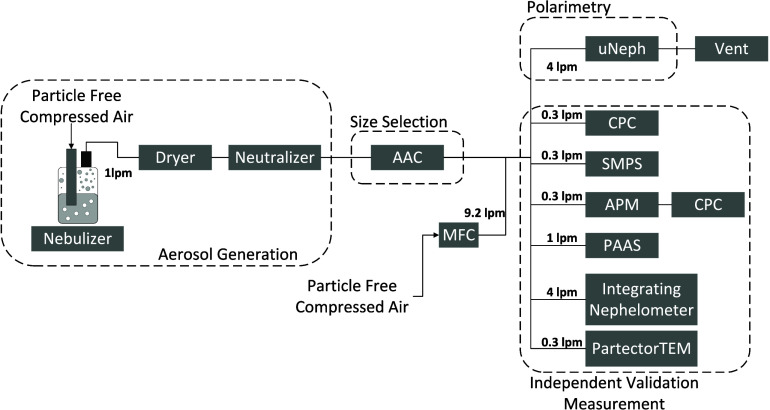
Experimental setup. uNeph: polar nephelometer; AAC: Aerodynamic Aerosol Classifier; MFC: Mass Flow Controller; CPC: Condensation Particle Counter; SMPS: Scanning Mobility Particle Sizer; APM: Aerosol Particle Mass analyzer; PAAS: Photo-Acoustic Absorption Spectrometer.

An aerodynamic aerosol classifier^64^ (AAC, Cambustion Ltd., UK) was used for size selection of the dried aerosol particles. The AAC operates by balancing centrifugal and drag forces to select particles of well-defined aerodynamic diameter (*d*_ae_). As a result, it produces truly unimodal size distributions that are narrow in terms of *d*_ae_, without any interference from larger multiply charged particles. Several sizes from the accumulation mode were selected for each test aerosol for subsequent measurements: specifically, aerodynamic diameters of 200, 250, 300, 400, 450, and 500 nm were selected at size resolution parameter (*R*_s_) 20. Each selected size was measured by the downstream instrumentation for around 30 min. The measurements reported hereafter all represent averages over these time periods. A complete list of measured aerosol properties for all aerosol samples is provided as a supplementary document.

#### 2.1.2 Polarimetric Measurements

A prototype of a laser imaging polar nephelometer called uNeph was used to measure phase function and polarized phase function of aerosol samples, i.e. of an ensemble of particles.^53^ The instrument measures the polarization resolved light scattering phase function at a wavelength of 532 nm, with high angular resolution between around 10° and 85° and between 95° and 170°. The calibration process for the uNeph is described in Moallemi et al. (2023).^53^ Figure S2 demonstrates the validity of uNeph calibration, with a root-mean-square error (RMSE) of 4.8% compared to the total scattering coefficient measured by an integrating nephelometer (see SI for details). Validation of angularly resolved light scattering data follows in Section 3.1.

In the Stokes formalism, light scattering by an ensemble of randomly oriented particles^65^ is described by phase matrix^53^ *F*(θ) shown in eq 1, which depends on microphysical properties of the particle ensemble such as shape, size distribution, and refractive index.^66^

1 $$F\left(\theta \right)=\left[\begin{array}{llll} F_{11}\left(\theta \right) & F_{12}\left(\theta \right) & 0 & 0\\ F_{12}\left(\theta \right) & F_{22}\left(\theta \right) & 0 & 0\\ 0 & 0 & F_{33}\left(\theta \right) & F_{34}\left(\theta \right)\\ 0 & 0 & -F_{34}\left(\theta \right) & F_{44}\left(\theta \right) \end{array}\right]$$

The phase function (PF), *F*_11_(θ), expresses the directional distribution of the radiance of the scattered light. The polarized phase function (PPF) stands for −*F*_12_(θ)/*F*_11_(θ), which expresses the relative degree of linear polarization of scattered light for unpolarized incident light.

#### 2.1.3 Independent Aerosol Property Measurements

Inspired by the proposal of Schuster et al. (2019)^39^ to validate the retrievals of polarimetric measurements with independent, parallel measurements, we employed a series of instruments to measure aerosol size, mass, absorption, and scattering in parallel with the uNeph, as shown in Figure 1. The main flow splitter, which distributes the size-selected and diluted sample to the common sampling line of all instruments, included a mixing chamber. A condensation particle counter (CPC 3775, TSI, USA) was used to measure the particle number concentration. A scanning mobility particle sizer (SMPS 3082, TSI, USA) provided the particle number size distribution as a function of the mobility diameter. An aerosol particle mass analyzer^67^ (APM 3601, Kanomax, Japan) was used to obtain the mass of the size selected particles (geometric mean). A photoacoustic absorption spectrometer^68^ (PAAS-4λ, schnaiTEC, Germany) provided the light absorption coefficient at 4 wavelengths (445 nm, 515 nm, 638 nm, and 785 nm). A 3-wavelength (461, 525, and 631 nm) integrating nephelometer (IN101, AirPhoton, USA) was used for the integrated light scattering coefficient and the hemispheric backscatter ratio. We also employed a TEM sampler (partectorTEM, NANEOS, Switzerland) to collect particles on copper grids for later TEM analysis. The independent instruments used for uNeph validation were calibrated and validated using standards and consistency checks as described further in Section S2 and demonstrated in Figures S3 to S5. The high level of agreement obtained with these consistency checks demonstrates the high quality of the independent data.

### 2.2 Black Carbon Morphology Modeling and Optical Forward Kernel

The mathematical description of fractal aggregates is given by the following equation:^69,70^

2 $$N_{s}=k_{f}{\left(\frac{R_{g}}{a_{0}}\right)}^{d_{f}}$$

Here, *N*_s_ is the number of primary particles, *a*_0_ represents the radius of the primary particles, *d*_f_ the fractal dimension, and *k*_f_ the fractal prefactor. *R*_g_ is the radius of gyration, which defines the spatial extent of the aggregate. For generating BC fractal aggregates of various morphologies, we used a software implementing the diffusion limit aggregation (DLA) algorithm,^71,72^ which simulates the formation of fractal structures through random, diffusive motion. The DLA code takes *N*_s_, *k*_f_, *a*_0_, and *d*_f_ as inputs and generates aggregates which additionally fulfill eq 2. These five parameters, including *R*_g_ through eq 2, constrain the aggregate shape with very limited freedom (if primary spherules are monodispersed).

MSTM (version 4.0, 2021)^73–75^ was used to simulate optical scattering of the fractal aggregates generated using the DLA algorithm. MSTM can be applied to arbitrary configurations of spheres located internally or externally to other spheres, with the only restriction being that the surfaces of the spheres do not overlap. The MSTM code simulates the phase matrix at various angles, as well as scattering, absorption, and extinction cross section for aggregates of spherical primary particles. For each set of fractal parameters (eq 2) we generated 50 particles using the DLA algorithm and applied MSTM in the random particle orientation mode to obtain an averaged phase matrix. Sensitivity analyses showed that, with considering random orientation, as few as approximately 5 particles are sufficient to obtain a statistically representative phase matrix (as shown in Figure S6). A considerably larger number of particles would be needed when not considering random orientation.

For optical simulation of spherical particles, we applied the Lorenz–Mie theory using the python package miepython v2.5.5 (12/1/2024).^76^ The code follows the procedure described by Wiscombe.^77^ The consistency of the Mie and MSTM codes in providing identical light scattering phase matrix elements was checked by considering single spheres.

### 2.3 Polarimetric Retrieval

The inversion consists of an aerosol model, an optical forward kernel, and an optimization algorithm. An aerosol model is a set of state parameters such size distribution or refractive index that define the optical properties of aerosols.^78^ The corresponding forward model, or kernel, uses these parameters to calculate key optical properties such as PF and PPF. The retrieval process involves minimizing the difference between simulated results from the forward model and real measurements,^79^ such as PF­(θ) and PPF­(θ) obtained from a polar nephelometer. The optimization algorithm adjusts the state parameters to achieve the best match. Once fitted, the state parameters can also be used to derive other relevant properties, such as particle volume concentration and absorption coefficient. In this study, we implemented two retrieval variants, i.e., uNeph-Mie for spherical particles and uNeph-MSTM for fractal aggregates.

The uNeph-Mie retrieval assumes homogeneous spheres with identical material properties (chemical composition) and a unimodal log-normal size distribution. Thus, the aerosol model has five state parameters. Two material parameters for the real and imaginary parts of the CRI at 532 nm wavelength (for uNeph measurements) and three size distribution parameters (geometric mean diameter, GMD, geometric standard deviation, GSD, and particle number concentration, *c*_num_). The forward kernel for this aerosol model was implemented by using the Mie code described in the previous section. The equation for calculating the residual between simulation and measurement is provided in the SI. Above aerosol state parameters are allowed to vary continuously and independently of each other. Optimization of this residual is done using the least-squares algorithms^80,81^ implemented through the Scipy package (v1.15.2).^82^

The uNeph-MSTM retrieval considers the shapes of the aggregates. Accurate retrieval of BC aggregate properties requires that the aerosol model used in the forward kernel strikes the balance between complexity and simplicity.^83^ We simplify aggregate morphology in the aerosol model by assuming identical spherical monomers, with each of them touching (but not overlapping with) one or multiple other monomers. In this case, three state parameters - volume equivalent diameter (*d*_ve_, defined as diameter of a sphere with identical volume as the particle), fractal dimension (*d*_f_), and monomer diameter (*d*_pp_) - are required to describe a single particle shape, in addition to assuming a fixed scaling prefactor (*k*_f_ = 1.593), as suggested by Wozniak et al. (2012),^72^ and requesting that eq 2 holds.

In the aerosol model for an ensemble of aggregates, we also considered the variability of the fractal dimension among individual particles. If an AAC is used to select a narrow size cut in terms of aerodynamic mobility diameter from a polydisperse aerosol sample consisting of particles with different fractal dimensions, then the selected particles vary considerably in fractal dimension and volume. The AAC selects particles according to *d*_ae_, which does not translate to well-defined fractal dimension of the selected particles for a polydisperse input aerosol. More compact particles with a smaller mass can have *d*_ae_ similar to that of less compact particles with a larger mass, due to negative covariance of *d*_ve_ and *d*_f_ for a fixed *d*_ae_. Drag force parametrizations available in the literature can be used to identify this covariance.^84^ Through our retrievals, we found a slope of around α = Δ*d*_ve_/Δ*d*_f_ = −180 nm). This value is approximately independent of the selected particle size (*d*_ae_). It is important to note that the value of α is specific to selecting particles by *d*_ae_ and to the BC aggregates studied here. For the retrieval, we assumed a triangular distribution of *d*_ve_ and replaced the size distribution parameters (GMD and GSD) with *d*_*f*_^center^, *d*_ve_^center^, and α. To account for the negative covariance of *d*_ve_ and *d*_f_, we used the parameter α for assigning *d*_f_ to each *d*_ve_. The width of the distribution, FWHM_*d*_ve__, was chosen such that extreme values correspond to *d*_*f*_^center^ ± 0.2. Overall, the aggregate aerosol model has two material parameters (RI_*n*_ as the real part of CRI and RI_*k*_ as the imaginary part of CRI), four shape and size parameters (*a*_0_, *d*_ve_^center^, *d*_f_^center^, *c*_num_), and two parameters for the particle ensemble (FWHM_*d*_ve__, α). The former six parameters were treated as free model parameters, FWHM_*d*_ve__ was prescribed, and α was held fixed at the preoptimized value of −180 nm. The phase function for the particle ensemble was obtained as an average of the individual particle phase functions weighted by the triangular distribution of *d*_ve_. We generated over 300000 particles with varying parameters as described in the SI. A least-squares method was used to minimize the residual between simulation and measurement. The residual was calculated using the same formula applied in the spherical aerosol model, and the particle number concentration was also allowed to vary freely.

## 3 Results

### 3.1 Retrieval Validation for Spherical Particles

First, we validate the polarimetric retrieval, specifically the uNeph-Mie retrieval, for spherical particles to demonstrate (i) performance of the uNeph instrument and (ii) feasibility of unbiased retrieval, if the aerosol model and forward kernel match all relevant features of the aerosol sample. We achieved good agreement within experimental errors between measured and fitted phase functions by applying the uNeph-Mie retrieval to quasimonodisperse aerosol samples. This is shown in Figure S7 for the examples of PSL and nigrosin particles with *d*_ae_ 600 nm, but it applies over the full range of tested sizes ranging from 150 to 1000 nm in *d*_ve_. The fitting was successful for both non-light-absorbing aerosols (PSL) and light-absorbing aerosols (nigrosin), within the experimental error estimated in a previous study.^53^

Furthermore, the uNeph-Mie-retrieved aerosol parameters show very good agreement with independently measured values, indicating that the retrieved properties are physically meaningful and unbiased. Specifically, the agreement for *d*_ve_ (GMD) and GSD is excellent for monodisperse PSL and nigrosin samples covering the range between 150 nm < *d*_ve_ < 1000 nm, with a RMSE of approximately 2.0% (GMD) of retrieval compared to independent data (Figure S8a and Figure S9a). This is well within uncertainty of the independent data, which are taken from the manufacturer’s NIST-traceable specifications (PSL) or derived from combined SMPS and AAC measurements (nigrosin; see SI for detail). The retrieved particle number concentration also agreed well with the CPC data (RMSE of 8.0%; Figure S8b), which is within the uncertainty of the CPC data. Accurate retrieval of number concentration and size goes along with good agreement for volume concentration (RMSE of around 7.5%; Figure S10a), where the independent volume concentration is derived from CPC and SMPS data (see SI).

The polarimetric retrieval also provides the CRI for the PSL and nigrosin samples (Figure 2). The reference CRI for PSL is the colored red star. The polarimetric retrieval for PSL across different sizes resulted in a mean value of 1.605 for the real part of the refractive index, with a RMSE of 0.0082, demonstrating good precision and accuracy compared to 1.599 reported in the literature.^54^ For the imaginary part of the refractive index, a mean value of 0.0028 was retrieved, which aligns with values (∼10^–3^) reported in other studies.^36,85^ This indicates that the uNeph-Mie retrieval, run without constraints on RI_*k*_, correctly identifies PSL particles as essentially nonabsorbing.

**Figure 2:**
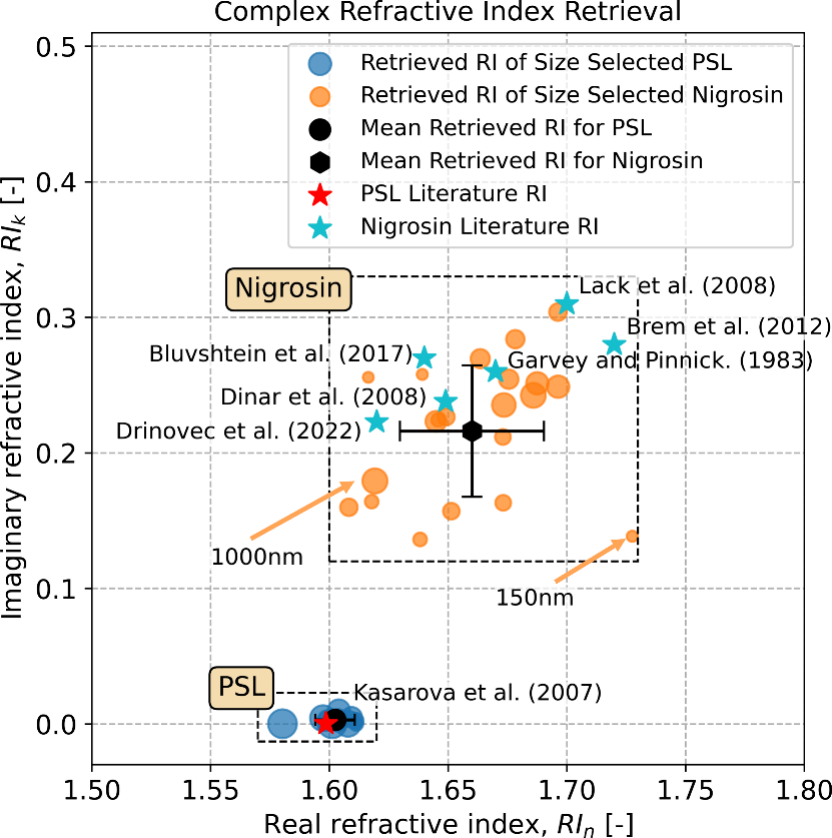
Retrieved CRI compared with literature data for monodisperse PSL and nigrosin aerosol samples of different sizes^54–61^ as indicated in symbol size ranging from 150 to 1000 nm. Both the real and imaginary parts of the refractive index were free parameters in either case.

For nigrosin, several studies have reported varying values for the CRI, using methods such as cavity ring-down aerosol spectrometer,^58,59^ spectroscopic ellipsometry,^61^ and scattering aerosol spectrometer.^57^ The discrepancies may be attributed to variations in optical properties between batches due to the nature of the particle production protocol.^61^ We used the mean value of these literature values of CRI = 1.655 + 0.260*i* as independent data. In Figure 2, the sizes of the blue and orange dots are proportional to the selected diameters of particles. The retrieved CRI is size independent, which aligns with the definition of the refractive index as an intrinsic optical property of the material. For nigrosin, the mean retrieved real refractive index was 1.645 with an RMSE of 0.026. The uncertainty in retrieval for nigrosin was slightly higher than for PSL, which aligns with theoretical analyses indicating that more absorbing particles tend to introduce greater errors in the inversion of the real refractive index.^36^ Slight nonsphericity can further add to uncertainty, due to spherical particle assumption in the optical calculations, though this effect likely is small. The mean retrieved RI_*k*_ for nigrosin was 0.221, with an RMSE of 0.058. The precision was lower than for the real part, which agrees with previous information content analyses for phase function data.^86^ Despite this slightly higher uncertainty, the retrieved value remains within the uncertainty bounds from other studies.

Feeding the retrieved aerosol parameters into the forward kernel also provided the total scattering and absorption coefficients of the aerosol sample. Accurate calibration of the uNeph usually results in accurate retrieval of the scattering coefficient (Figure S2), given the retrieval essentially is a fit to measured PF and PPF data. By contrast, the quality of the retrieved absorption coefficient depends critically on the accuracy of all aerosol parameters, including CRI. The absorption coefficient of the PSL samples was below the lower detection limit of the PAAS. For nigrosin, the retrieved absorption coefficient falls well within the uncertainty range of the PAAS (RMSE of 6.1%; Figure S10b). Combining data from an integrating nephelometer and PAAS, the retrieved single scattering albedo (SSA) for both PSL and nigrosin also falls well within the uncertainty range (RMSE of 7.7%; Figure S9b).

Above retrieval results demonstrate that the polarimetric retrieval on uNeph measured PF and PPF can provide accurate and precise aerosol properties for unimodal spherical aerosol samples without applying any a priori constraints beyond selecting the aerosol model parameters. This statement applies for all retrieved and derived aerosol properties, i.e., volume concentration, size distribution, and optical properties including the imaginary refractive index, light absorption coefficient and SSA. Notably, we allowed the RI_*k*_ to be a completely free parameter in the retrieval, rather than fixing it as a prescribed constant as previously done in some polar nephelometer retrievals.^38^ Earlier theoretical and numerical studies using information content analysis or deep learning approaches demonstrated retrievability of these aerosol parameters including absorption coefficient,^34,86^ nevertheless, it is valuable to demonstrate it with real measurement data. Successful validation of the uNeph-Mie retrieval for the case with spherical particles lays the foundation for testing BC polarimetric retrieval in the next section.

### 3.2 Retrieval of Bare Black Carbon Aggregate Properties

#### 3.2.1 Comparison of Measured and Fitted Phase Functions

Here we assess the polarimetric retrieval of aerosol properties for bare black carbon aggregates. Measured and fitted phase functions of size-selected BC aggregates are shown in Figure 3 (four examples) and Figure S11 (two more sizes). Using the uNeph-Mie retrieval (blue curves) does not provide a good match with the measurement (black curves) for both phase function and polarized phase function. Systematic deviations occur for PF of larger particles and θ > ∼150°, and more so for PPF across all angles and sizes. By contrast, when accounting for the aggregated morphology using the uNeph-MSTM kernel, a good fit within experimental uncertainty can be achieved for both PF and PPF at all sizes (red curves).

**Figure 3:**
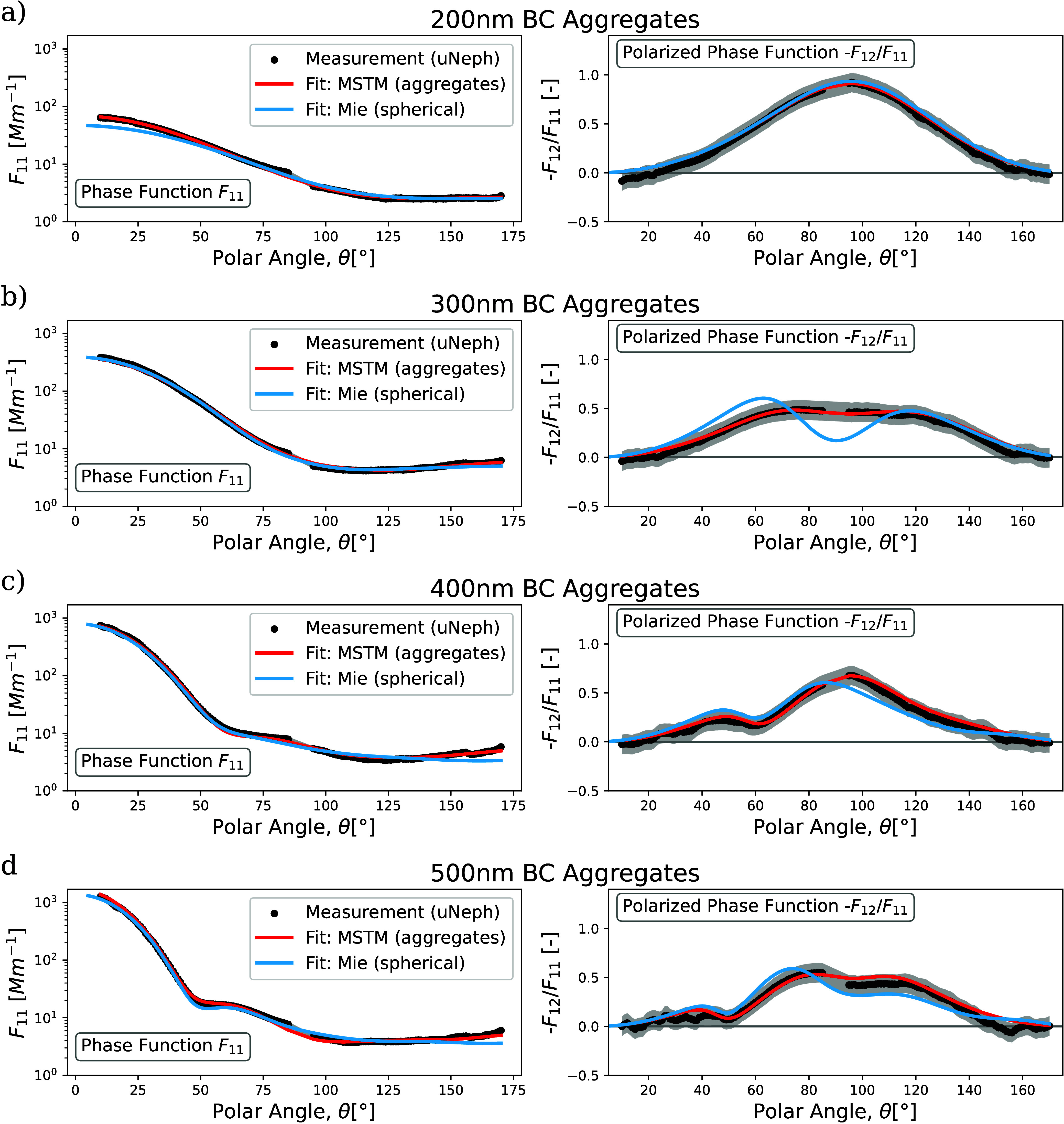
(a–d) Phase function and polarized phase function of aggregates: example measurement and fit. Gray shading indicates the estimated measurement error taken from a previous study.^53^

Aggregate shape has distinct effects on the shape of PF and PPF. In theory, the PF resembles that of a sphere larger than the volume equivalent sphere, i.e., exhibiting stronger forward scattering. In contrast, the PPF of an aggregate resembles that of a sphere with smaller volume; i.e., the bell-shape characteristic for Rayleigh scatterers is retained up to a larger particle volume. Previous measurements of BC aggregates from premixed flames,^87^ aircraft exhaust,^43^ or commercial carbon samples^88^ have demonstrated this feature. Such a combination of PF and PPF is not achievable for unimodal, homogeneous spherical aerosols. Therefore, previous studies using Mie theory also failed to adequately match both the PF and PPF simultaneously.^44,89^

#### 3.2.2 Comparison of the Accuracy of the Mie- and MSTM-Based Retrievals

A fundamentally important question is whether a better fit of the measured phase function, as achieved with the MSTM kernel compared to the Mie kernel (Figure 3), also results in improved aerosol property retrieval. Figure 4 presents a comparison of several retrieved aerosol properties to independent measurement data. Using the spherical aerosol model with a Mie kernel results in systematic bias for all properties, which is as high as ∼70% for the absorption coefficient or ∼300% for the volume concentration. Considerable systematic differences between measured and fitted phase functions visible in Figure 3 show that the uNeph-Mie retrieval does not fully reproduce the measurement. The result in Figure 4 demonstrates that this can result in substantial bias in the retrieved aerosol properties. The disagreement between fit and measurement in Figure 3 indicates that the Mie kernel may not be appropriate, hence indicating that retrieved parameters must be interpreted with caution. The sign and magnitude of the bias shown in Figure 4 for the uNeph-Mie retrieval are not expected to be robust nor more generally applicable. For example, altering calculation of the fit residuals, provided in eq S1, could change the uNeph-Mie retrieval result. Consequently, the difference in uNeph-Mie retrieval performance between particle sizes *d*_ae_ = 200 and 250 nm likely is a random result.

**Figure 4:**
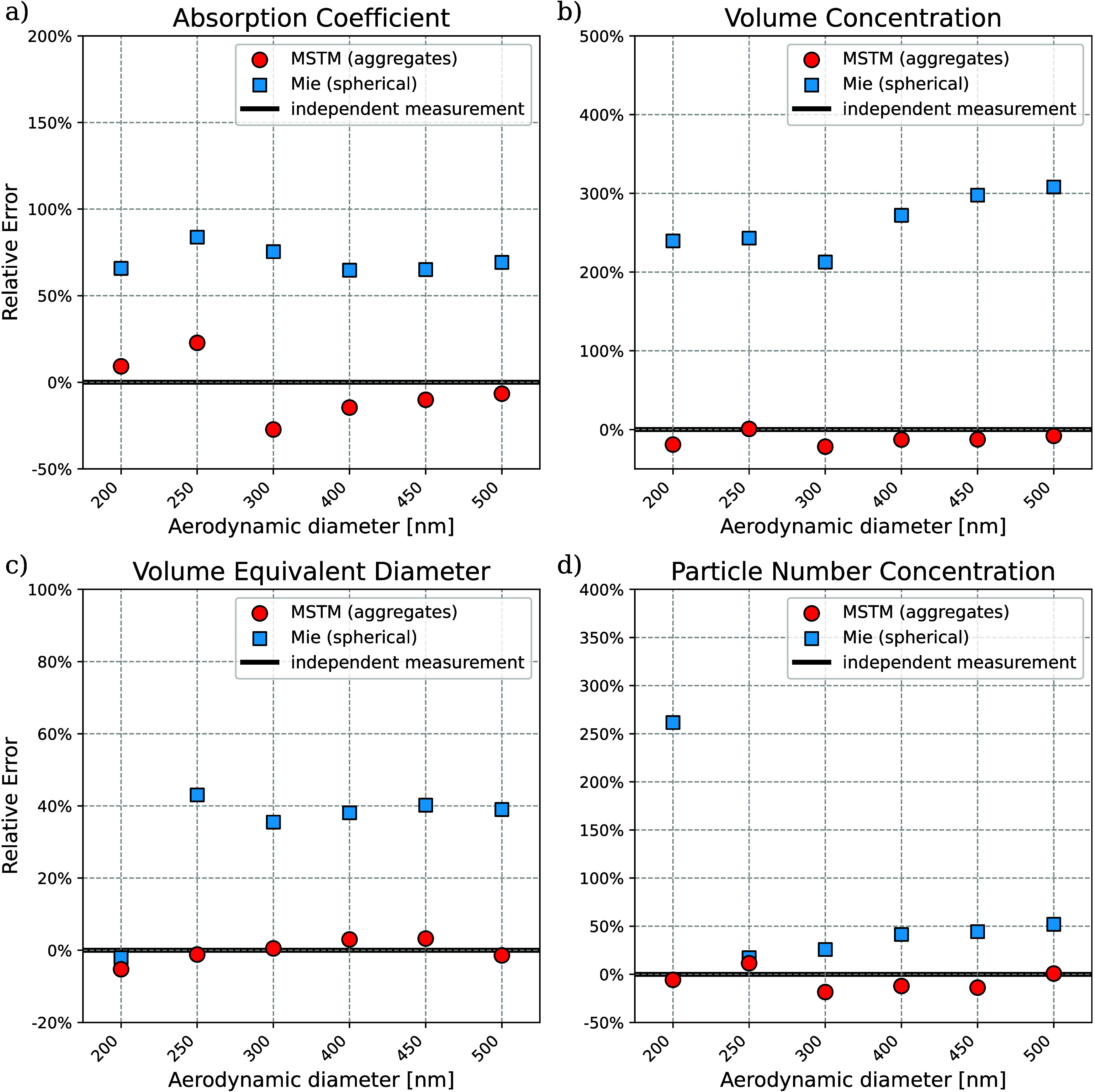
Performance of different optical kernels: MSTM versus Mie for the aerosol parameter a) absorption coefficient, b) volume concentration, c) volume equivalent diameter, and d) particle number concentration.

Considering the aggregate shape and using the MSTM kernel provides good agreement with independent data for the BC aggregates. Relative errors, averaged over the different sizes, are −4.4% for the absorption coefficient, −12.2% for volume concentration, −0.2% for volume equivalent diameter, and −6.3% for particle number concentration. Notably, for the sample with *d*_ae_ = 200 nm the uNeph-MSTM retrieval also achieves substantially smaller error in retrieved parameters than the uNeph-Mie retrieval (Figure 4), for which the Mie retrieval achieves nearly as good a fit to measured PF and PPF as the MSTM retrieval (Figure 3). We will thus focus on the MSTM results in the following.

#### 3.2.3 Precision of the MSTM-Based Retrievals

The purpose of the retrieval is to determine the physically meaningful aerosol properties. This goal requires, in addition to achieving a good fit of PF and PPF, also a reasonable precision, i.e., an unambiguous solution with limited uncertainty of the best fit parameters. Therefore, we assess the variability among the top 50 combinations of aerosol parameters (out of 2 million), i.e., those 50 combinations of parameter values achieving the lowest residuals between simulated and measured PF and PPF for each aerosol sample. The top 50 threshold was chosen such that their fit residuals approximately remain within the range of values expected based on uNeph measurement uncertainties previously quantified by Moallemi et al. (2023).^53^ The top 50 retrievals fall within a contiguous range in the multidimensional parameter space for each size-selected BC sample (not shown), which indicates a well-defined global minimum and an unambiguous retrieval result. Hence, we illustrate precision of retrieved and derived aerosol parameters by means of a box-and-whisker plot (Figure 5). High precision is observed for the real part of the refractive index, the volume-equivalent diameter, and the two morphology parameters fractal dimension and monomer diameter. The morphology parameters have a very small variation for each size, indicating that they can serve as equivalent parameters to cover the influence of particle shape in the uNeph-MSTM kernel. Only moderate precision is observed for the imaginary part of the refractive index (see Figure 5). This qualitatively agrees with findings of a previous information content analysis, which indicated higher uncertainty for the imaginary part of the refractive index retrieved from PF and PPF.^86^ This uncertainty also affects other parameters, foremost number concentration, though to a lesser extent. Precision of the retrieved absorption coefficient is better than that of the imaginary part of the refractive index. Additionally, the precision of volume concentration is slightly better than that of number concentration, as the PF responds more directly to volume than to number concentration. Retrieval precision is somewhat poorer for the 300 nm data point. However, this likely is a random result rather than a general relationship between the particle size and retrievability.

**Figure 5:**
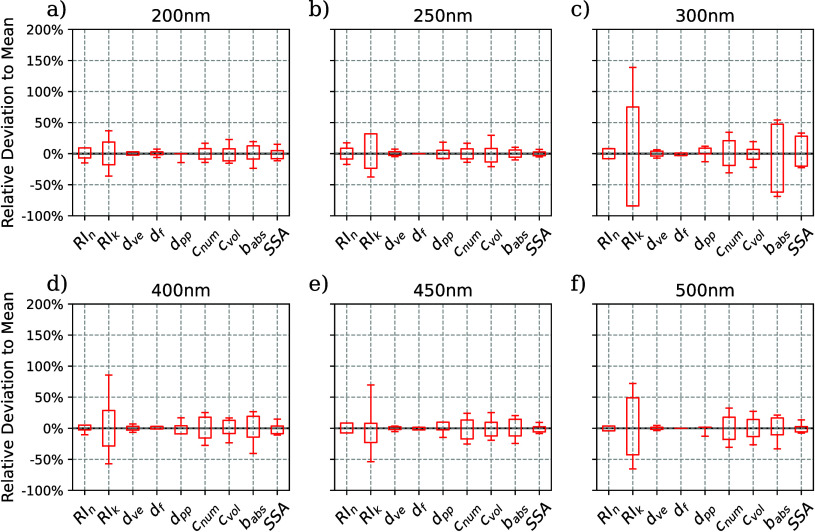
(a–f) Precision of retrieved and derived aerosol parameters (statistics of the top 50 ranked state parameter combinations relative to their mean value). Results for different aerodynamic diameters are shown in separate panels. Boxes show the interquartile range (IQR), and the whiskers extend to the farthest data point lying within the 10th or 90th percentile (*d*_pp_: primary monomer diameter; *c*_vol_: volume concentration; *b*_abs_: absorption coefficient).

#### 3.2.4 Accuracy of the MSTM-Based Retrieval

Next, we compare the uNeph-MSTM retrieval results with independently measured properties of the size-selected BC aggregates to assess the accuracy of the retrieved aerosol parameters in more detail. Figure 6 shows the relative error of the four retrieved properties for each selected particle size. Results from single size retrievals, shown in red, agree with independent measurements (i.e., parallel measurement of the same quantity with different instruments) within retrieval precision and measurement uncertainty. It can therefore be concluded that the MSTM kernel explains the shape of measured PF and PPF (Figure 3) and provides accurate retrieval of the BC aggregate properties, as shown in Figure 6. This includes volume concentration, particle volume equivalent diameter, SSA, and absorption coefficient, which poses a challenge in aerosol polarimetry. Figure S12 shows that retrieval of the number concentration also works for the special case of unimodal size-selected samples studied here. It can be expected that retrieval of volume concentration also works for polydisperse size distributions as all particle sizes with a relevant contribution to volume concentration also make a relevant contribution to phase function. By contrast, retrieval of total number concentration is expected to become uncertain, if the tail of a broad size distribution extends down into the ultrafine size range (diameter <100 nm), where the scattering cross sections drops steeply compared to larger particles.

**Figure 6:**
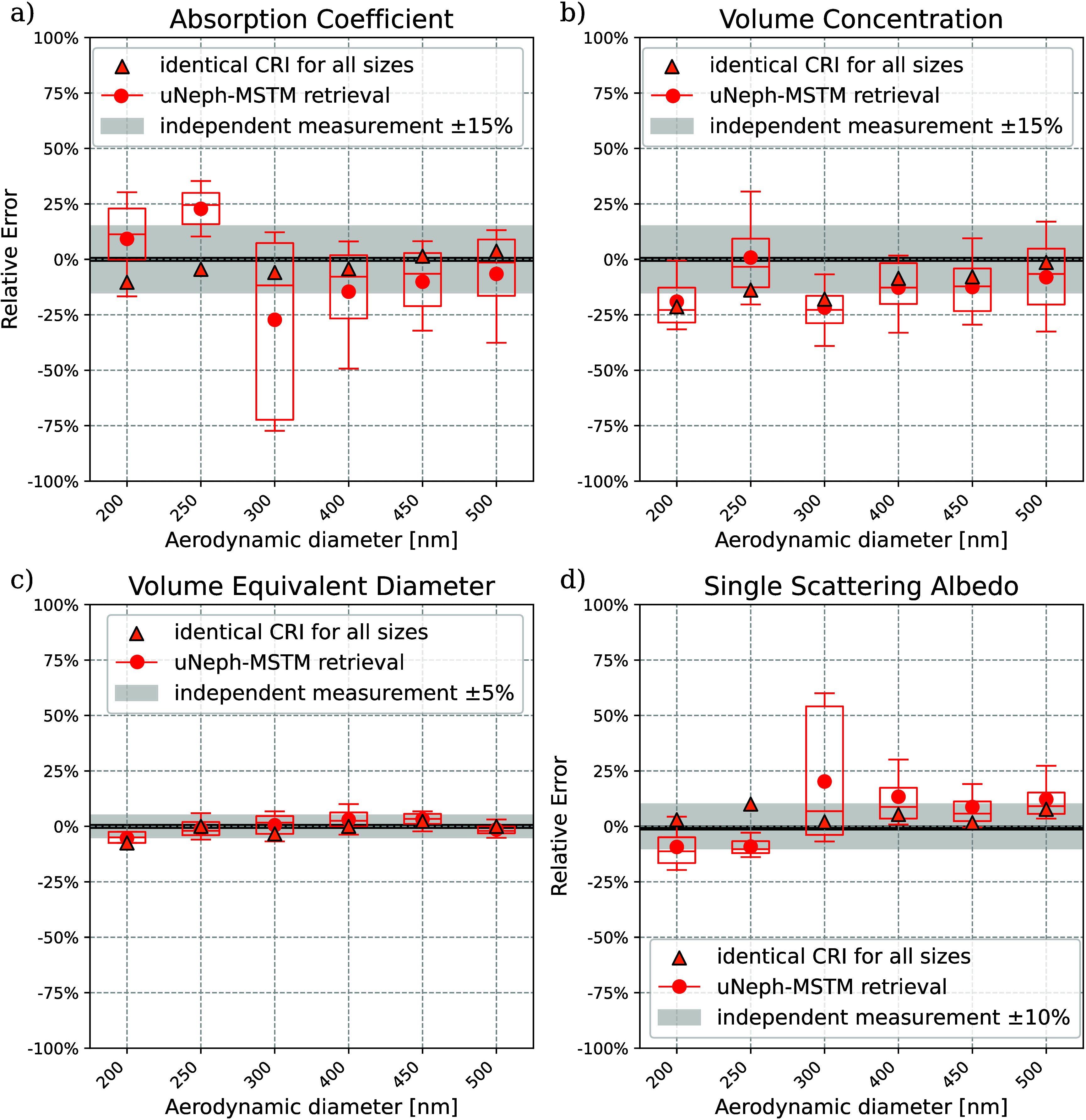
Benchmarking uNeph-MSTM retrieval results (red) for size-selected BC aggregates against independent measurements (black) of a) absorption coefficient, b) volume concentration, c) volume equivalent diameter, and d) single scattering albedo. Box and whiskers indicate retrieval precision; the gray shading indicates uncertainty of the independent data. Orange markers indicate uNeph-MSTM retrieval results, enforcing identical complex refractive index for all sizes.

To further investigate whether the residual uncertainty is random or systematic, we performed a combined retrieval across all sizes simultaneously. This approach leverages the assumption that the CRI, a material property, is independent of the particle size. Specifically, we retained the refractive index as a free parameter in the combined retrieval under the constraint that it must be identical for all sizes. In this way, the refractive index we finally retrieved is 2.53 + 0.77i, which is in reasonable agreement with the range of values (2–3, 0.5–1) reported in the literature.^90,91^ Other retrieved aerosol parameters come to excellent agreement with independent data for all sizes (orange data points in Figure 6): Average relative errors of −9.9% for volume concentration, −0.4% for particle volume equivalent diameter, −0.6% for absorption coefficient, and −2.8% for number concentration are well within the uncertainty of the independent data. Furthermore, this agreement achieved with the above constraint is better for all sizes than retrieval results without this additional constraint (orange data points in Figure 6). This indicates that residual uncertainty of single size retrievals is for the most part of a random rather than systematic nature. This result further corroborates the excellent performance of the aggregate aerosol-model and MSTM kernel combination for these bare BC aggregates, i.e., that it accurately simulates their optical behavior.

## 4 Discussion

Our study is the first one, to the best of our knowledge, making use of polarimetric retrievals with an optical kernel considering fractal aggregate shape to demonstrate agreement between measured and simulated PF and PPF of BC aggregates. A previous study, which also used an MSTM forward kernel to consider the shape of BC aggregates, found good agreement between measured and simulated PF. However, the simulated PPF values around a 90° polar angle were systematically lower than measured.^51^ To further investigate the source of this discrepancy, Yazici et al. (2023)^92^ performed forward simulations for the same data set which additionally considered necking between the primary spherules. However, this only resulted in limited improvement and did not remove systematic bias.^92^ We hypothesize that this discrepancy is more likely attributed to systematic errors in the aerosol state parameters used in the forward calculations, rather than owing to an oversimplified shape (e.g., perfectly spherical monomers without necking) or limited accuracy of the MSTM kernel. In the study by Jia et al. (2020),^51^ the BC particle production process involved collection and reaerosolization steps, which may lead to compaction of some aggregates. Then they used mobility analysis, TEM and drag force parametrizations to indirectly determine particle volume and fractal-like dimension. So, the input parameters fed to the optical forward kernel are somewhat uncertain. More so, potential effects of variability in compactness between individual particles on the phase function remained unconsidered in the forward simulations. Indeed, it can generally be expected that aggregate compactness varies considerably between individual particles.^93^

The study by Kelesidis et al. (2020)^94^ is the only previous work, to our knowledge, that achieved quantitative agreement between measured and simulated polarized phase functions for BC aggregates. They sampled the aerosol at a fixed height directly above the flame, which is expected to result in well-defined fractal dimension with limited particle-to-particle variability.^95^ Discrete Element Modeling (DEM) was used to describe particle shape while considering polydispersity of primary particles and necking using independent mobility, particle mass, and TEM analyses. DDA was then used to calculate the phase function. This approach using DEM coupled to DDA made it possible to accurately simulate the number or primary particles, effective density, polarized phase function, and absorption coefficient. However, considering this level of detail for particle shape is impossible in retrieval algorithms, as it creates too many free parameters.

Our results indicate that a simplified uNeph-MSTM approach is sufficient to reproduce PF and PPF and light absorption of size-selected BC aggregates. While this finding applies for an ensemble of particles that differ slightly from each other, it may not apply for the differential scattering cross section of a single particle. Kelesidis et al. (2020) emphasized the importance of considering monomer polydispersity and necking in reproducing polarimetric measurements and light absorption,^94^ whereas previous work by Jia et al. (2020) also explored various monomer polydispersity cases and found no significant improvement.^51^ Our results also show that neither polydispersity nor necking is required to reproduce measured optical (PF, PPF, absorption coefficient, and SSA) and physical properties (volume concentration and volume equivalent diameter). However, our simplified aggregate representation does not provide the correct effective density values when scaling laws are applied for the mass-mobility relationship. Altogether, we interpret the results of these studies as follows. Detailed description of particle morphology, i.e., considering polydispersity of monomers and necking, is relevant to get all particle physical and optical properties right. Using a simplified aggregate representation with monodisperse spherical monomers combined with MSTM is sufficient to obtain accurate results for volume equivalent diameter, volume concentration, PF, PPF, absorption coefficient, and SSA. Retrieved number of monomers, monomer radius, and fractal dimension must be taken as “effective parameters” which may differ from actual values. Further assessing this against independent measurements is rather difficult as quantification of the monomer radius is method-dependent for realistic aggregate morphologies.^96^ Furthermore, as the idealized morphology is only an approximation of the actual morphology, it may not be possible to use the effective model parameters (number of monomers, monomer radius, and fractal dimension) obtained with the polarimetric retrieval to infer the mobility diameter of the particles. This statement also applies in the opposite direction; i.e., it may not be possible to use the combination of mobility and mass or TEM measurements to infer the effective model parameters for running the MSTM in forward direction, as Jia et al. (2020) tried to do without success.^51^ Furthermore, our results show that considering particle-to-particle variability of (effective) fractal dimension is important to get the optical properties right, at least in the special case of a unimodal aerosol obtained by AAC size selection from a polydisperse bare BC aerosol sample. Figure S13 illustrates that the measured phase functions are much smoother than phase functions obtained by assuming identical fractal dimension for all particles. Considering variability of particle compactness likely becomes less important for polydisperse BC aggregates, as polydispersity also has a smoothing effect.

Previous experimental studies demonstrated that using the MSTM forward kernel outperforms Mie theory for integrated optical parameters.^49^ Here we show that the MSTM kernel also provides accurate phase function simulation, thus enabling accurate retrievals of aerosol properties including SSA for bare BC. While this shows that there is no fundamental obstacle, the SSA retrieval challenge does become much more difficult for atmospheric aerosols due to their complexity, e.g., particle-to-particle variability of size, shape, composition, and mixing state. This requires simplifications in the aerosol, including the common assumption of spherical shape. Schuster et al. (2019)^39^ generated a wide range of different aerosol mixtures in the laboratory, performed in situ measurements of PF and PPF, and applied the Generalized Retrieval of Aerosol and Surface Properties (GRASP) algorithm to retrieve aerosol properties in a similar way as the AERONET retrievals applied to sun photometry. They found systematic differences in SSA compared to independent measurements. The spherical shape assumption may contribute to this bias; however, other simplifications like the mixing state in the aerosol model certainly also play a role.

Accurate SSA retrieval from, e.g. AERONET data , remains a challenge.^16,97,98^ The above results suggest that there is no fundamental limitation in retrieving SSA of BC aggregates from polarimetric data. It is expected that this also holds for more atmospherically relevant BC aggregates with lower fractal dimension and smaller primary spherules (see Figure S1 and associated discussion). In addition, our results suggest that the capability to acquire polarization resolved measurements of the present PACE and upcoming 3MI satellite missions may be an opportunity for better SSA retrievals. Therefore, we suggest further laboratory and field studies to investigate whether considering nonspherical shapes of BC particles and their mixing state (e.g., BC attached to or embedded in nonabsorbing particulate matter) can potentially improve SSA retrieval in more atmospherically relevant samples or whether other simplifications in the aerosol model hinder any improvement for complex mixtures.

## Supplementary Material





## Notes

Additional information on samples, instrument validation, retrieval method validation, and further analysis of forward kernel performance (PDF)
Supporting data tables providing results of independent measurements and retrievals (XLSX)
