# Supplementary material for: Evaluating and Improving Light Absorption Retrievals of Black Carbon Using In Situ Polar Nephelometry
Source: Environ Sci Technol. 2025 Sep 25;59(39):21076–89. doi: 10.1021/acs.est.5c05919 (PMC12509318; doi:10.1021/acs.est.5c05919)
Supplement: Supplementary file 1 [file es5c05919_si_001.pdf]

Supporting Information

Evaluating and Improving Light Absorption Retrievals of Black Carbon Using In Situ Polar Nephelometry

Qizhi Xu[1], Barbara Bertozzi[1], Robin Lewis Modini[1], Benjamin Tobias Brem[1], Thomas Müller[2], Baseerat Romshoo[2], Claudia Mohr[1,3] and Martin Gysel-Beer[1,]

[1]PSI Center for Energy and Environmental Sciences, 5232 Villigen PSI, Switzerland

[2]Leibniz Institute for Tropospheric Research, 04318 Leipzig, Germany

[3]Department of Environmental Systems Science, ETH Zürich, 8006 Zürich, Switzerland

KEYWORDS: light scattering, polarimetry, aerosol property retrieval, black carbon aggregate, Multi-Sphere T-Matrix, light absorption, phase function

SUMMARY

Additional information on samples, instrument validation, retrieval method validation and further analysis of forward kernel performance. 16 pages including 13 figures (Figures S1-S13).

1. Aerosol Sample

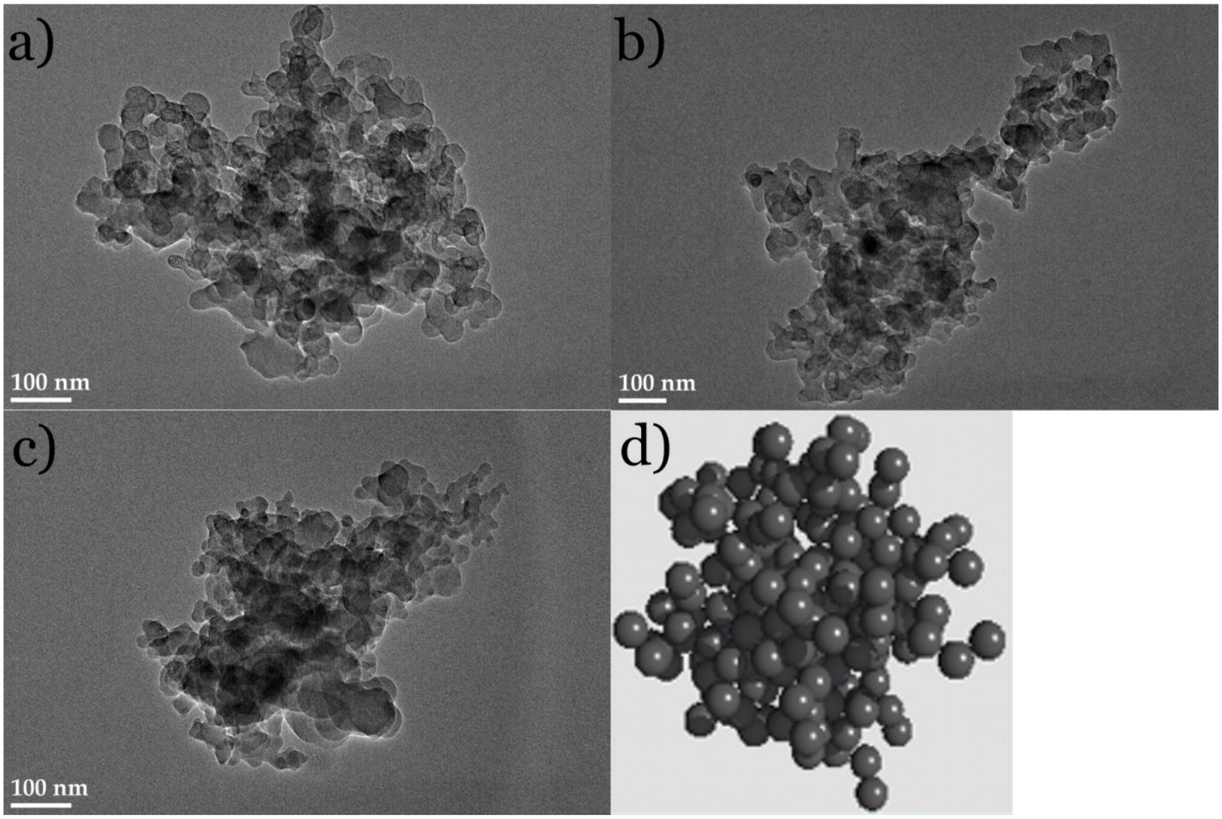

Figure S1. a), b), c) TEM images of fullerene soot (size selected for an aerodynamic diameter of 300nm); d) Fractal particle obtained from DLA modelling (with the retrieved fractal dimension).

2. Instrument Validation

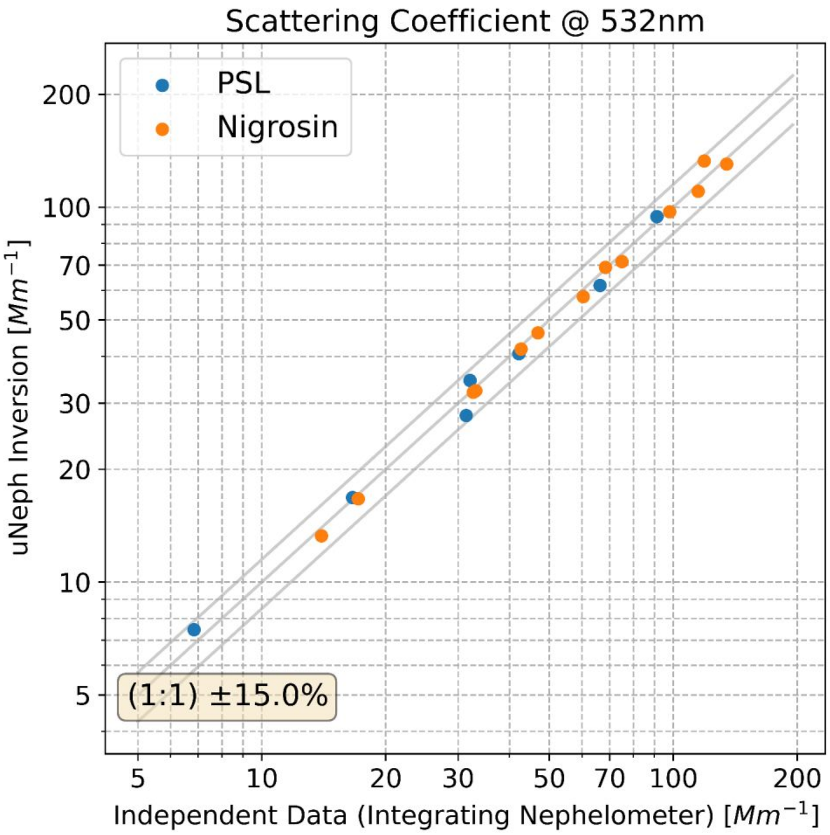

Figure S2. uNeph versus independent data: total scattering coefficient for particles ranging from 150 nm to 1000 nm volume equivalent diameter. Independent data are from an AirPhoton IN101 integration nephelometer. Truncation correction has been applied to the IN101 data with considering its actual illumination function and the known phase function shape of the monodisperse spherical aerosol samples. The relative RMSE is 5.5%. We interpolated the integrating nephelometer data using a power law, i.e. assuming a constant Angström exponent between two wavelengths below and above the wavelength of the uNeph. The interpolation error is expected to be small as one wavelength of the integrating nephelometer is close to the wavelength of the uNeph.

a)

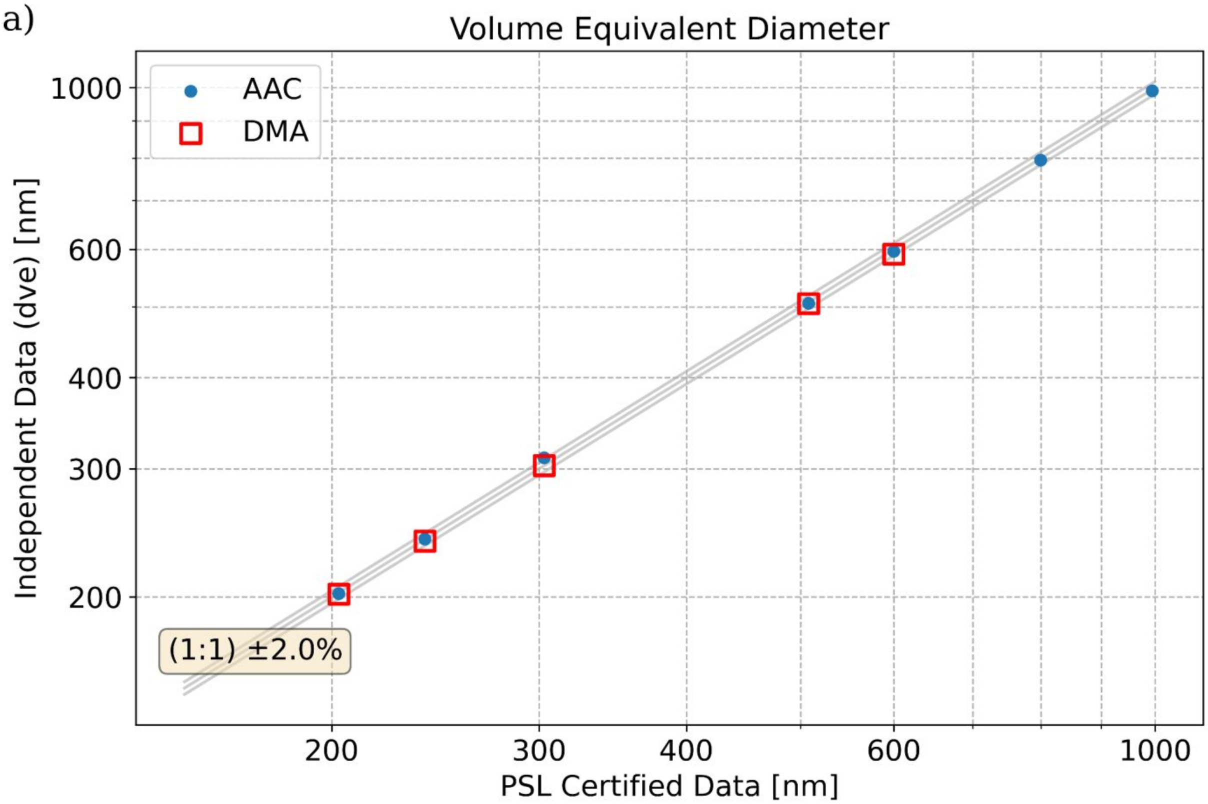

Volume Equivalent Diameter
AAC
DMA
Independent Data (dve) [nm]
1000
600
400
300
200
(1:1) ±2.0%
200
300
400
600
1000
PSL Certified Data [nm]

b)

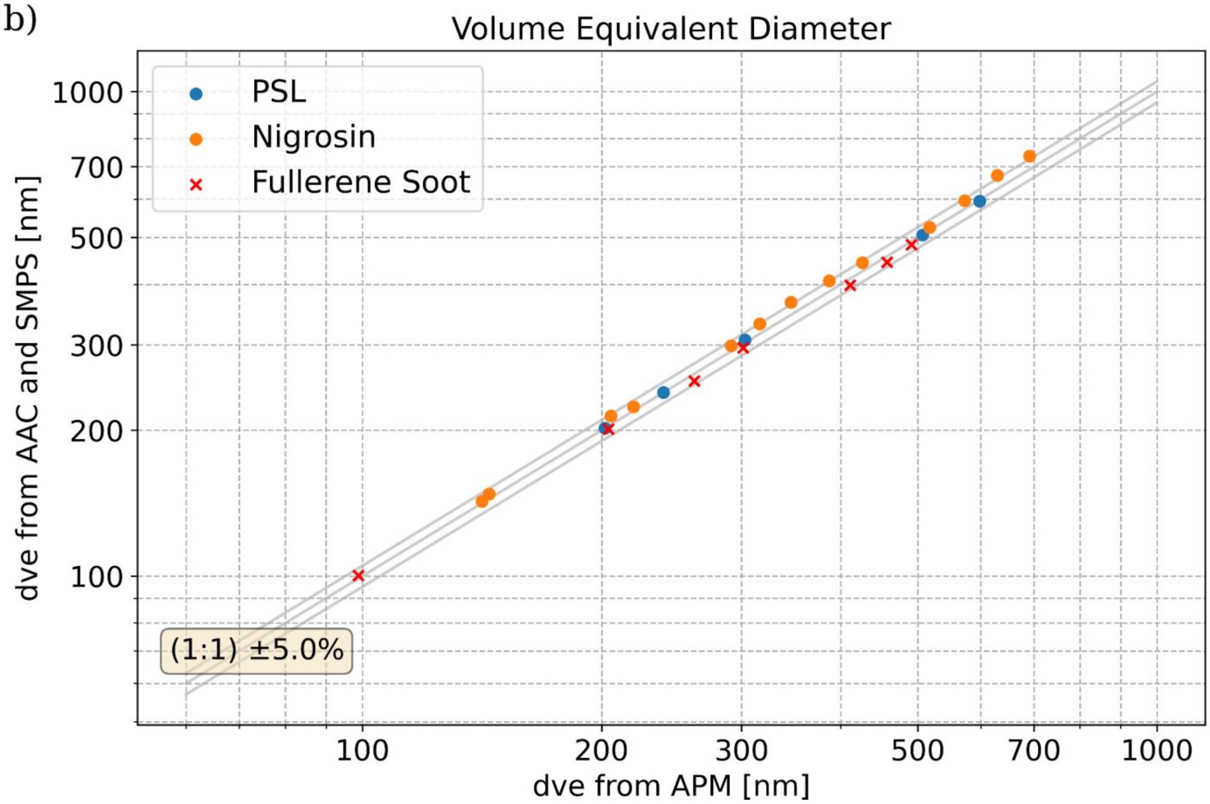

Volume Equivalent Diameter
PSL
Nigrosin
Fullerene Soot
dve from AAC and SMPS [nm]
1000
700
500
300
200
100
(1:1) ±5.0%
100
200
300
500
700
1000
dve from APM [nm]

Figure S3. Instrument validation. d_{ve}: volume equivalent diameter. a) the relative RMSE is 1.0% for AAC, 0.6% for SMPS. b) the relative RMSE is 3.3%.

Figure S3 shows validation of AAC, SMPS and APM. The APM directly provides particles mass. The combination of AAC, which selects particles by relaxation time (product of particle mass times particle mobility), and SMPS, which measures particle mobility, also provides particle mass. Both approaches work for any particle shape. The only caveat is that interference of multiply charged particles in APM and SMPS measurements can be eliminated, which is readily possible for such monodisperse samples. Particle mass measured by either APM or AAC and SMPS was converted to void-free volume equivalent diameter using material densities of 1050 kg m^{-3} for PSL, 1650 kg m^{-3} for nigrosin, and 1800 kg m^{-3} for fullerene soot. Figure S3a validates AAC and DMA against certified diameter of PSL size standards. Figure S3b demonstrates consistency of AAC, SMPS, and APM measurement within 5% relative error and independent of particle shape.

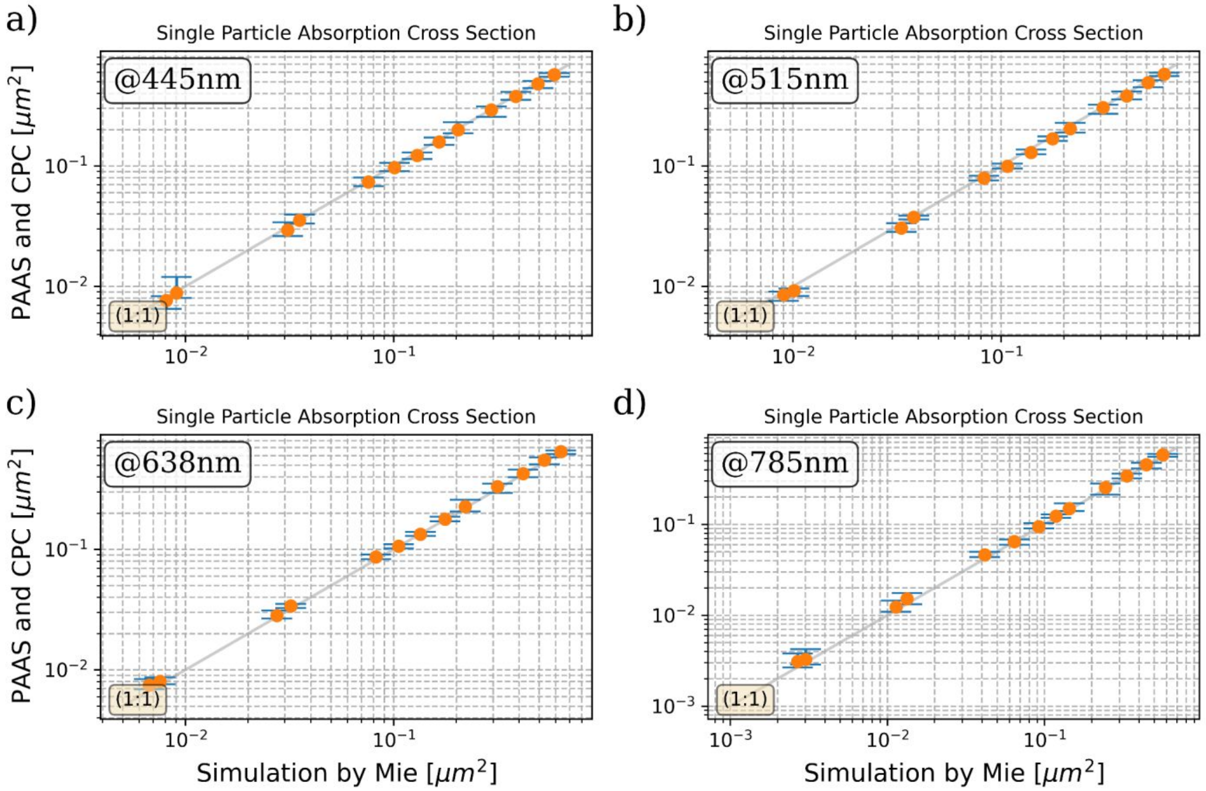

Figure S4. Validation of PAAS (four wavelength) and CPC against Mie simulations for nigrosin particles in terms of single particle absorption cross-section. Dividing absorption coefficient (PAAS) by number concentration (CPC) provides measured single particle absorption cross-section shown on the y-axis. Mie calculations were constrained with measured particle diameter and refractive index taken from the literature (see Section 3.1 in the main text). Data points cover AAC-selected samples with volume equivalent diameters ranging from 150 nm to 1000 nm. The relative RMSE is around 5.3% averaged over all wavelengths. We interpolated the PAAS data using a power law, i.e. assuming a constant Angström exponent between two wavelengths below and above the wavelength of the uNeph. The interpolation error is expected to be small as one wavelength of the PAAS is close the wavelength of the uNeph.

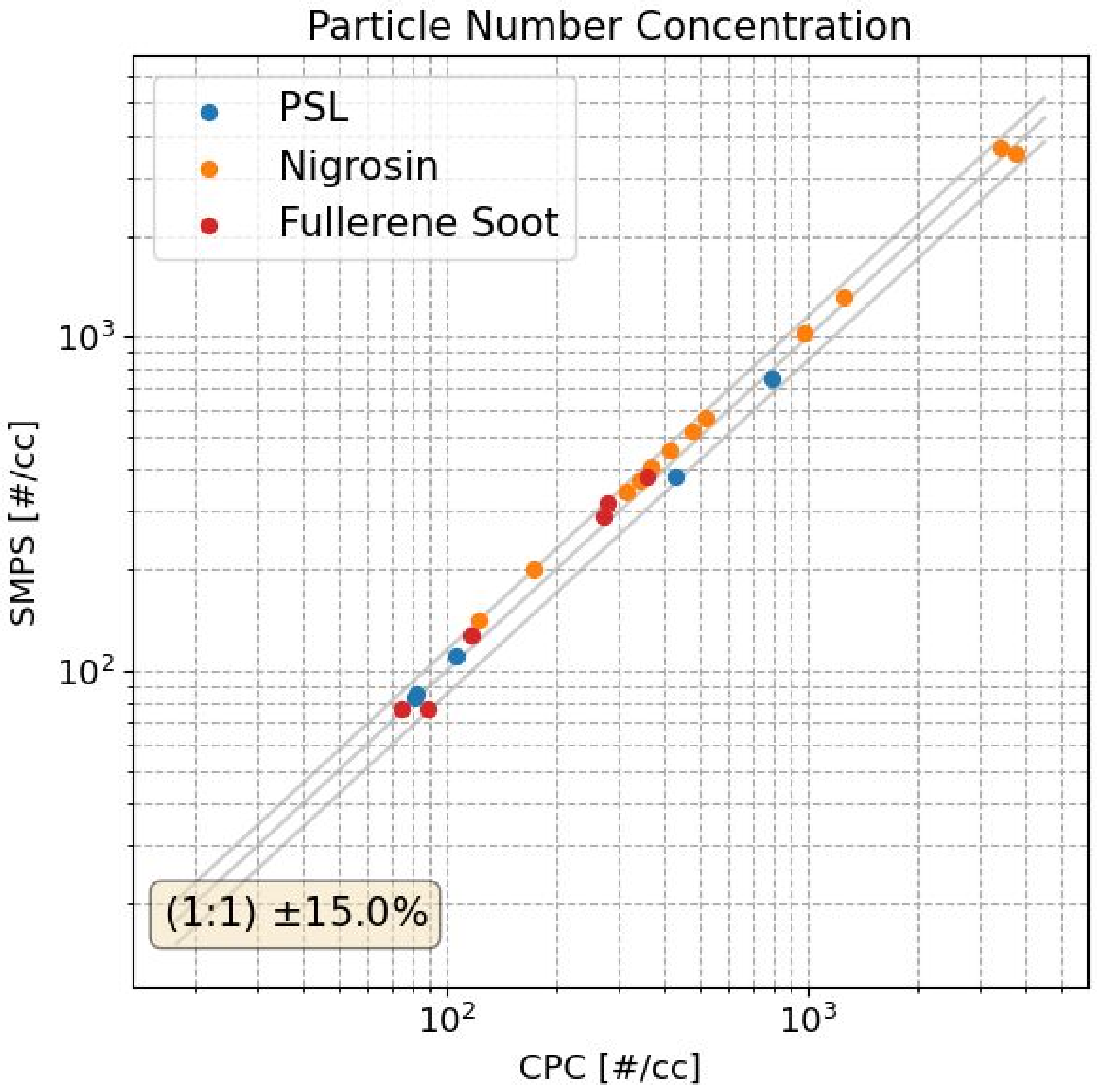

Figure S5. Validation of SMPS and CPC for total particle number concentration. They agreed with a relative RMSE of ~8.5%.

3. Simulation Validation

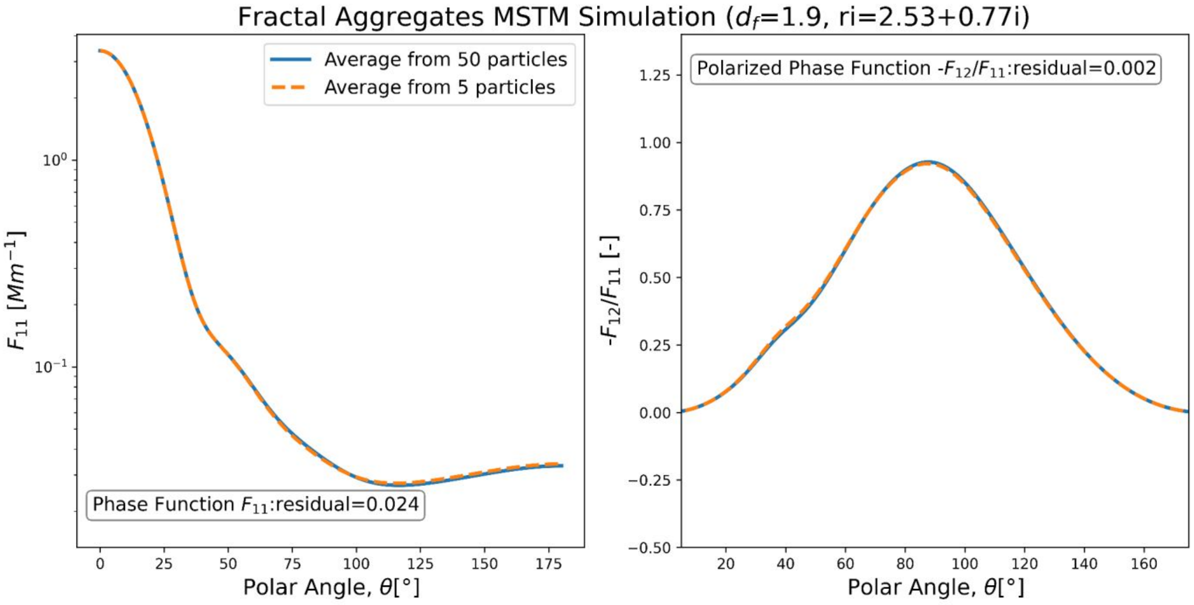

Figure S6. Example demonstrating that the average of 5 particles is sufficient to obtain statistically representative phase matrix elements that are virtually identical to the average of ten times more randomly generated particles. This statement applies for MSTM simulations with computing the randomly oriented average phase matrix for each of these particles. Alternatively, one could simulate a considerable larger number of particles with only considering a single orientation for each of these particles.

4. Additional information on measurement uncertainty estimation

Table S1 provides an overview of main aerosol parameters along with corresponding measurement methods and uncertainty estimates. The SMPS and AACE were validated using PSL size standards, which leaves an uncertainty of ~3% for mobility diameter and aerodynamic

diameter, respectively. For the CPC, the manufacturer states an uncertainty of 10%. The volume equivalent diameter is inferred from mobility diameter, aerodynamic diameter and material density, which results in a combined uncertainty of ~5%. Particle volume concentration requires number concentration in addition to volume equivalent diameter, thus resulting in ~15% uncertainty. The total scattering coefficient has an uncertainty of ~5% (for the truncated values assessed in Fig. S1). The calibration uncertainty of the aerosol absorption coefficient is estimated to be ~15%. Propagating the uncertainty of scattering and absorption coefficient results in ~10% uncertainty for SSA for the SSA range covered in this study.

Table S1. Estimation of measurement uncertainty

Variable	Instrument(s)	Additional parameter	Calibration method	Uncertainty estimate
Mobility diameter d_{mob}	SMPS		PSL size standard	3%
Aerodynamic diameter d_{aero}	AAC		PSL size standard	3%
Particle number concentration C_{num}	CPC		n.a.	10%
Volume equivalent diameter d_{ve}	SMPS and AAC	Material density	n.a.	5%
Particle volume concentration C_{vol}	SMPS, AAC, and CPC	Material density	n.a.	15%
Total aerosol scattering coefficient b_{sca}	Airphoton Integrating Nephelometer		Gas Calibration	5%
Aerosol absorption coefficient b_{abs}	PAAS		Nigrosin	15%
Aerosol single scattering albedo	Airphoton Integrating		n.a.	10%

SSA	Nephelometer and PAAS			

5. Additional information on optimized residual in retrieval algorithm

The residual between simulation and measurement is calculated as follows.

$$\sum_i \left\{ \left(log_{10} PF_{sim}\left(\theta_i; \overrightarrow{param}_{model}\right) - log_{10} PF_{meas}(\theta_i) \right)^2 + \left(PPF_{sim}\left(\theta_i; \overrightarrow{param}_{model}\right) - PPF_{meas}(\theta_i) \right)^2 \right\} \quad \text{(S1)}$$

As PF can vary by orders in magnitude as a function of θ, we follow common practice in aerosol polarimetry to log-transform PF for the calculation of residuals to provide equal weighting for all angles.

6. Additional information on parameter range and gridding for the MSTM retrieval.

Simulating the optical properties of particles with a volume-equivalent diameter of around 500 nm takes over half an hour, making it impractical to explore the parameter space in a continuous manner, as can be done with Mie theory. Hence, we implemented the uNeph-MSTM kernel in a discretized parameter space and prepared a pre-computed lookup table. Fractal particles morphologies were generated using the DLA model, setting the fractal dimension between 1.8 and 2.8 in increments of 0.1. The monomer diameter ranged from 35 nm to 90 nm in steps of 5 nm. The number of monomers varied from 10 to 1000, with a step size of 5 for aggregates with fewer than 200 monomers, and 25 for aggregates with more than 200 monomers. This approach resulted in volume-equivalent diameters approximately evenly varying from 50 nm to 900 nm. The real

part of the complex refractive index was set from 1.9 to 2.7 in steps of 0.2, and the imaginary part ranged from 0 to 2 with a step of 0.2. MSTM was then used for optical simulations of the generated particles. By deploying the code to a high-performance computing cluster, we were able to simulate light scattering for over 300,000 particles with varying parameters, which covers the possible range for our lab measured samples.

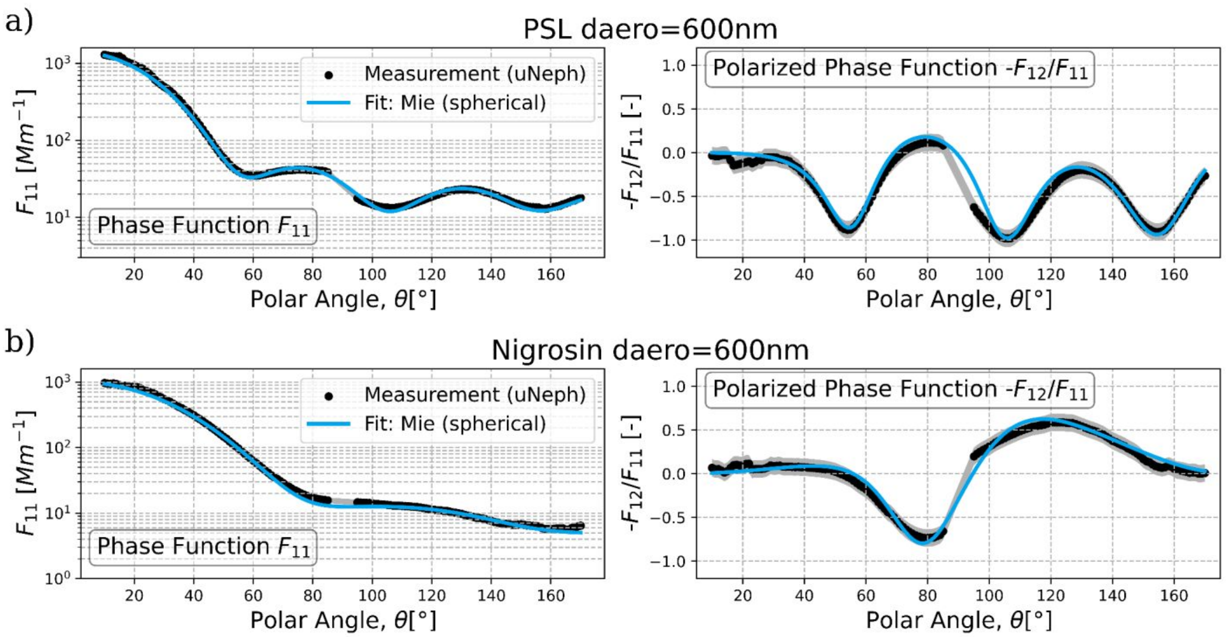

Figure S7. Examples of retrieved phase functions of PSL and nigrosine using uNeph-Mie retrieval (d_{aero}: aerodynamic diameter), gray shading indicates the basic estimate of uNeph measurement error (1σ) taken from Moallemi et al. (2023).

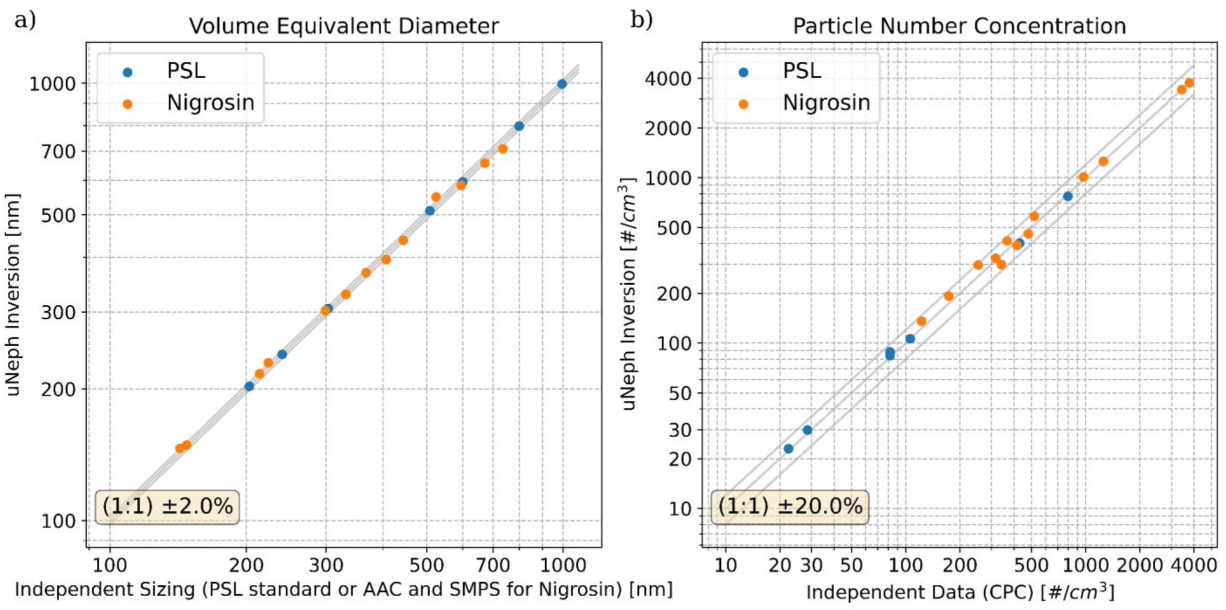

Figure S8. Validation of uNeph-Mie retrieval result for spherical particles against independent data: a) volume equivalent diameter and b) particle number concentration.

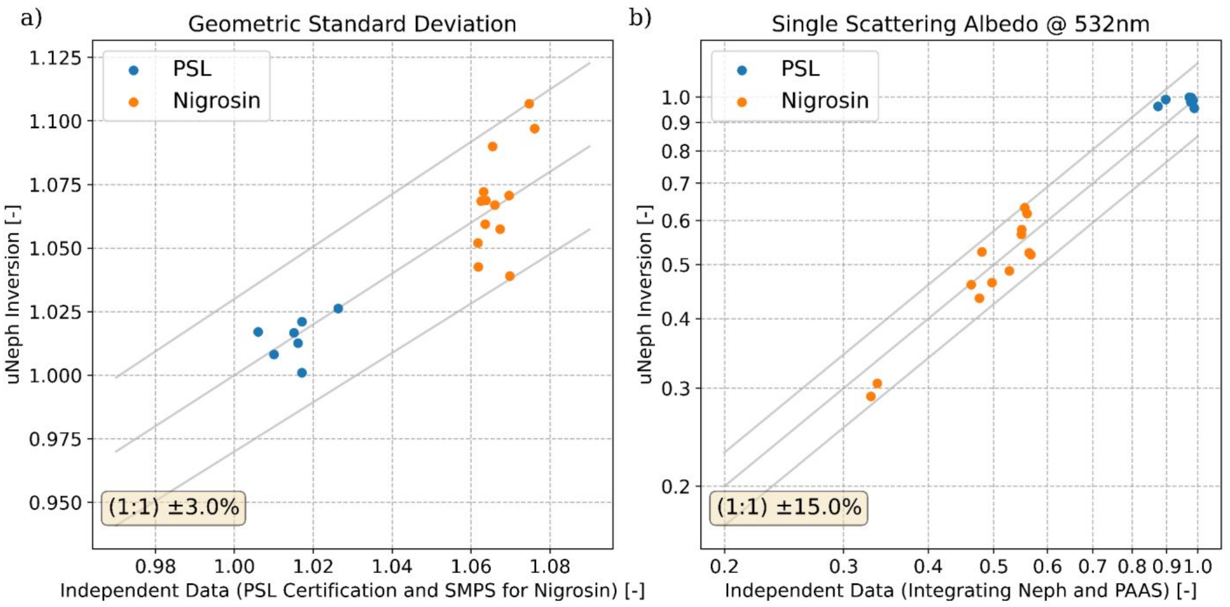

Figure S9. Validation of uNeph-Mie retrieval result for spherical particles against independent data: a) geometric standard deviation (GSD) and b) single scattering albedo. For PSL spheres, which have very small GSD, the independent GSD data is taken from specifications of the size

standards as the finite resolution of the SMPS would introduce a high bias. For nigrosin samples, which have a larger GSD, the independent GSD data is taken from the SMPS measurement. Truncation correction has been applied to the integrating nephelometer data.

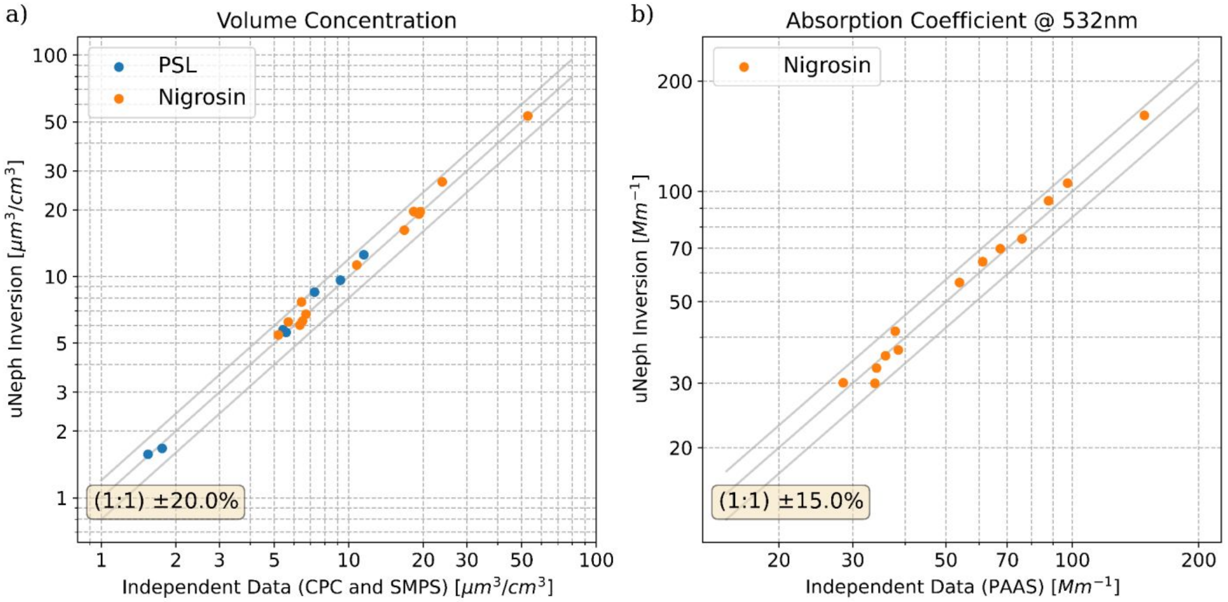

Figure S10. Validation of uNeph-Mie retrieval result for spherical particles against independent data: a) volume concentration and b) absorption coefficient.

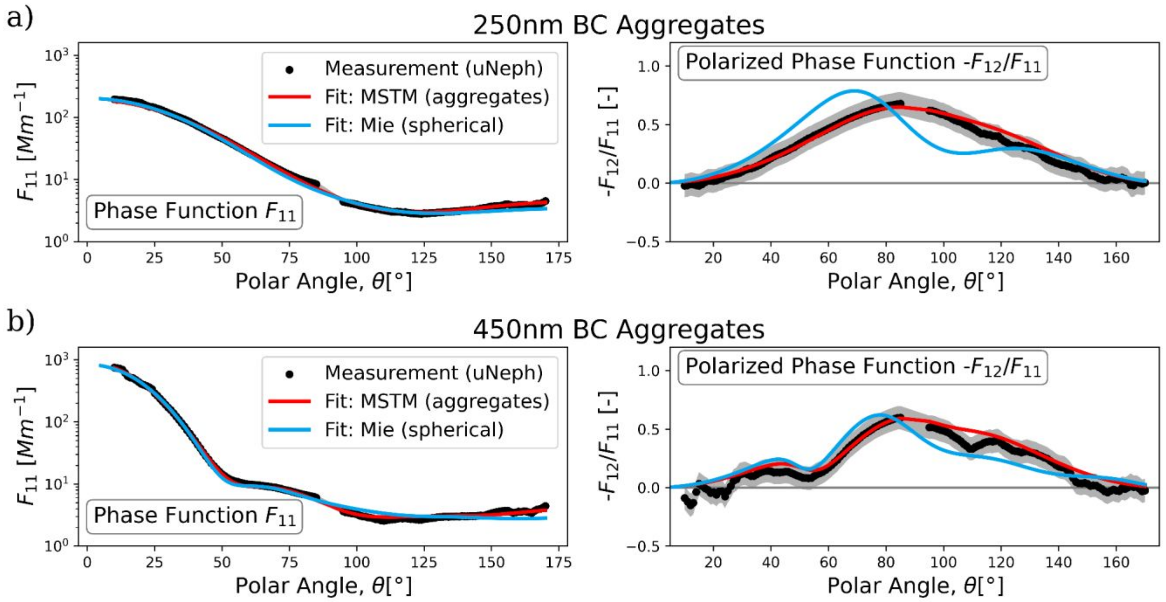

Figure S11. Polarized phase function of aggregates. Same as Figure 3 but for different particle sizes (aerodynamic diameter).

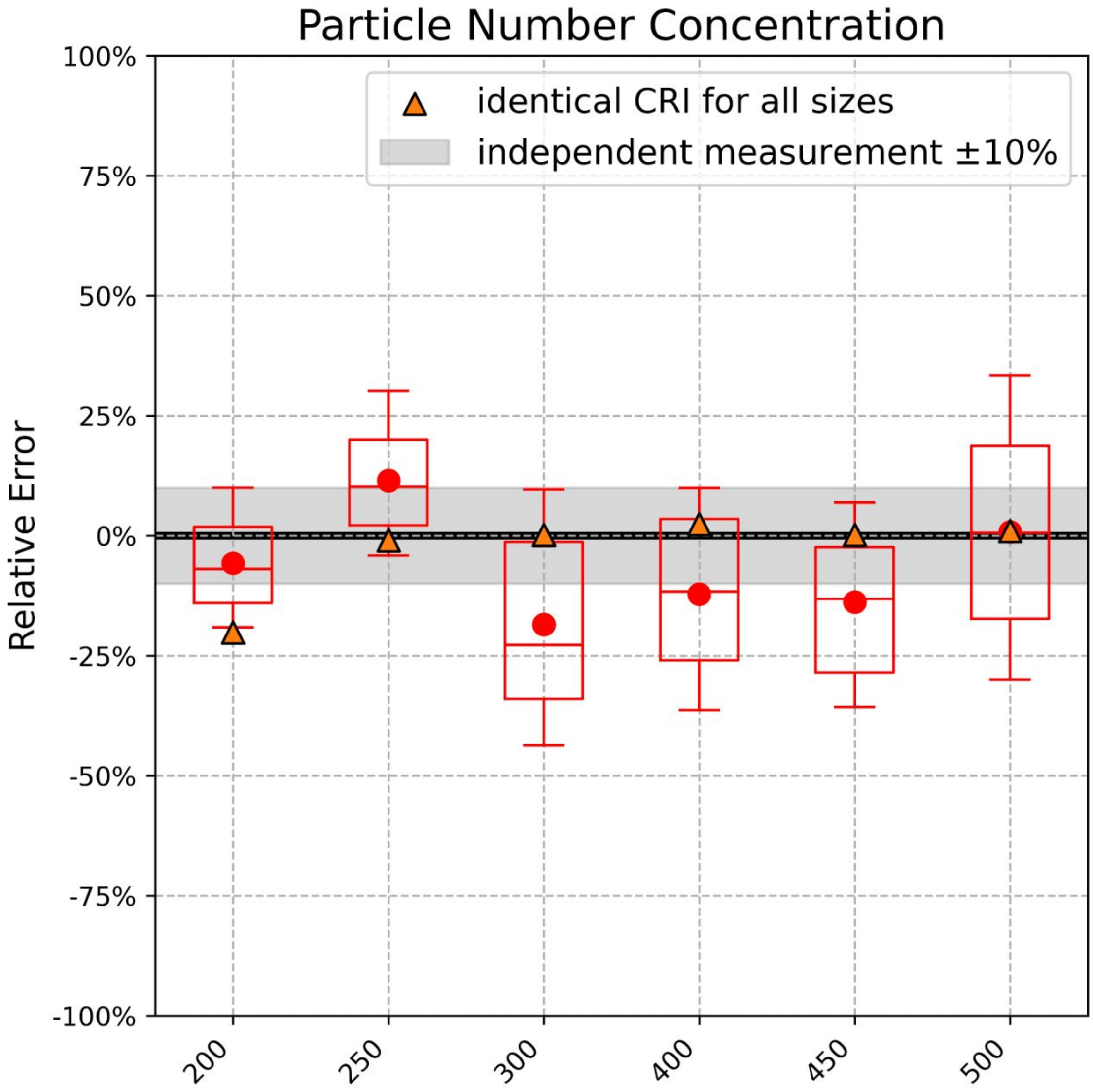

Figure S12. Benchmarking uNeph-MSTM retrieval results (red) for size-selected BC aggregates against independent measurements (black) for particle number concentration. Box and whiskers indicate retrieval precision, the grey shading indicates uncertainty of the independent data.

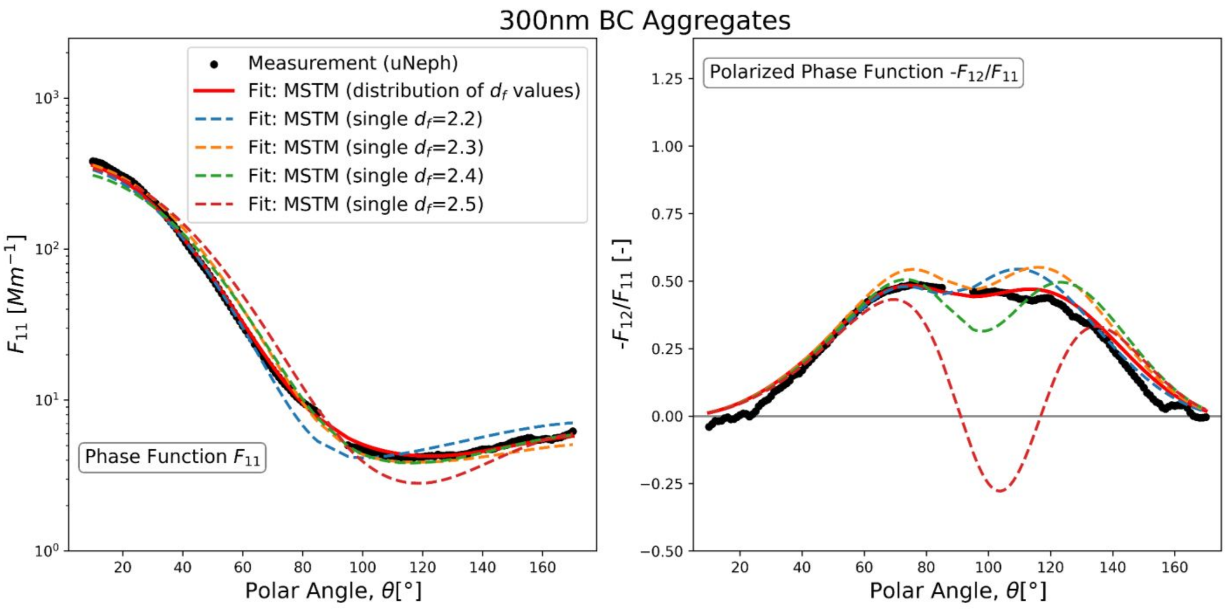

Figure S13. This figure demonstrates that it is only possible to reproduce the measured phase matrix elements (black lines) with considering an ensemble of particles covering a range of fractal dimensions (red solid lines). By contrast, just allowing for one discrete fractal dimension does not provide a good fit. The dashed lines show the best fit achieved with fixing the fractal dimension at four specific values. The measured phase function is considerably smoother than all these phase functions with a single fractal dimension.
